# Exploring Childhood Lower Urinary Tract Symptoms (LUTS), Urinary Tract Infections (UTIs) and the Microbiome—A Systematic Review

**DOI:** 10.3390/life15050730

**Published:** 2025-04-30

**Authors:** Mauro Van den Ende, Laure Van de Steen, Karel Everaert, François Hervé, George Bou Kheir

**Affiliations:** Department of Urology, ERN eUROGEN Accredited Centre, University Hospital Ghent, 9000 Ghent, Belgiumgeorge.boukheir@uzgent.be (G.B.K.)

**Keywords:** microbiome, lower urinary tract symptoms (LUTS), urinary tract infection (UTI), childhood

## Abstract

Pediatric lower urinary tract symptoms (LUTS) are influenced by age and coexist with nocturnal enuresis (NE) and bladder-bowel dysfunction (BBD). Urinary tract infections (UTIs) are common and linked to LUTS, though the causal relationship remains unclear. This systematic review aims to analyze microbiome alterations in pediatric LUTS and UTIs. Methods: A systematic review was conducted following PRISMA guidelines. PubMed, Embase, and CINAHL databases were searched for studies analyzing gut and urinary microbiomes in pediatric patients with LUTS and UTIs. Quality assessment was performed using the QUADOMICS checklist. Results: Nine studies published between 2018 and 2024 were included; seven out of nine studies employed prospective designs. Six hundred nineteen patients (44.3% pathology groups, 55.7% controls) were analyzed, with microbiome sequencing performed on stool samples in four studies and urine samples in five studies. UTIs and BBD were associated with reduced alpha diversity and distinct bacterial compositions, while beta diversity analyses revealed distinct clustering of microbiome compositions between affected and healthy groups. The gut microbiome of UTI patients showed alterations in *Actinobacteria* and *Proteobacteria* abundance, while voiding dysfunction (VD) was linked to the presence of *Fusobacterium nucleatum*, *Clostridium difficile*, and *Bacteroides clarus* without significant VDSS correlation. Conclusion: This systematic review reveals microbial alterations in pediatric LUTS and UTIs, with lower urinary diversity in UTI patients and sex-specific differences post-puberty. Microbiome-based interventions may offer novel therapeutic strategies for LUTS and UTIs.

## 1. Introduction

Childhood lower urinary tract symptoms (LUTS) and urinary tract infections (UTIs) are common clinical concerns with significant implications for lifelong urinary health and quality of life [1]. LUTS encompasses a spectrum of storage and voiding dysfunctions, including urinary incontinence, urgency, frequency, hesitancy, and dysuria, often presenting in conjunction with nocturnal enuresis (NE) and bladder and bowel dysfunction (BBD) [2]. UTIs, among the most prevalent infections in children, have a multifactorial etiology that includes anatomical abnormalities, functional bladder disorders, and immune responses [3]. While the bidirectional relationship between LUTS and UTIs remains incompletely defined, it is widely accepted that LUTS may predispose children to recurrent infections, while UTIs themselves can exacerbate urinary dysfunction [4,5].

Advancements in sequencing technologies have transformed the study of microbiota, providing detailed insights into microbial communities within both the gut and urinary tracts. Next-generation sequencing (NGS) approaches, such as 16S ribosomal RNA (rRNA) gene sequencing, have facilitated high-resolution profiling of microbial taxa and functional pathways. With the declining cost of sequencing, NGS is becoming the preferred method for microbiota characterization, enabling a comprehensive analysis of microbial diversity and metabolic function. These technological advances have highlighted the role of microbial metabolites as key mediators of host physiology, including immune responses, bladder function, and inflammation regulation. Urinary microbiome dysbiosis, namely, reduces the protective mechanism of healthy urinary microbiota, allowing uropathogen colonization and causing potential LUTS or UTIs [6]. Commensal urinary bacteria help maintain appropriate immune responses, while dysbiosis can lead to altered inflammatory states in the urinary tract [7]. Emerging evidence also suggests that gut dysbiosis may play a role in UTI development. Alterations in the gut microbiota during infancy could influence immune system maturation and autonomic nervous system coordination, potentially increasing the risk of UTIs [8]. However, the extent to which these microbiome variations contribute to LUTS or UTIs in children remains unclear, and further mechanistic investigations are needed to elucidate causal relationships [9].

Improvements in sample collection via suprapubic aspiration or sterile transurethral catheterization and microbiome analysis have further refined the study of microbial niches along the urinary and gastrointestinal tracts [10]. Non-invasive urine sampling has enabled urinary microbiome characterization, yet intra- and inter-individual variability poses challenges in defining reference microbial profiles [11]. Identifying microbial biomarkers associated with LUTS and UTIs in children could facilitate early detection, risk stratification, and targeted interventions to optimize urinary health outcomes from a lifelong perspective [12].

Although potential interactions between vaginal and urinary microbiomes in relation to LUTS and UTIs exist, advanced sequencing techniques have demonstrated the urinary microbiome to be independent from vaginal microbiota [10,13]. Associations between specific urinary bacteria and urinary urgency incontinence (UUI) have been found without corresponding changes in vaginal microbiota [14,15]. As bladder and bowel dysfunction often coincides in the pediatric population, the brain-bladder-gut axis needs to be examined in children with LUTS and UTIs [16]. Therefore, the aim of this systematic review is to summarize evidence regarding alterations in both the gut and urinary microbiomes in relation to LUTS and UTIs in the pediatric population, identifying key microbial patterns and potential pathways that may contribute to urinary dysfunction. By integrating microbiome analysis with clinical urological outcomes, this research aims to provide novel insights into microbial influences on pediatric urinary health and inform future targeted interventions.

## 2. Materials and Methods

This systematic literature review was conducted in adherence to the Preferred Reporting Items for Systematic Reviews and Meta-Analyses (PRISMA) guidelines. The 2020 PRISMA checklist [17] was followed, and it can be found in Table A1. The protocol was registered with the international prospective register of systematic reviews (PROSPERO CRD420250655637).

The screening process was conducted using Rayyan (http://rayyan.qcri.org) and Silvi**^®^** Version 1.7.2 (http://app.silvi.ai) to streamline study selection and data management. Rayyan was used for the primary screening of titles and abstracts from three databases (CINAHL, PubMed, and Embase), facilitating title and abstract selection and duplicate removal. Following the initial screening, Silvi.AI, a semi-automated AI-based platform, was used for the full-text eligibility assessment. This tool assisted in storing full-text PDFs and streamlining the review process by integrating controlled AI-based content analysis [18].

### 2.1. Inclusion Criteria

This review included microbiome analyses of gut and/or urine samples from a population <18 years old with LUTS and UTIs. Both gut and urine samples were analyzed for microbiome results with rRNA sequencing. Individual LUTS were included, as well as UTIs diagnoses: lower urinary tract symptoms, urinary bladder diseases, nocturia, urinary incontinence, nocturnal enuresis, bed wetting, urinary tract infections, pyelonephritis, cystitis, overactive bladder, urinary urgency, urge incontinence, urinary frequency, and voiding dysfunction.

### 2.2. Exclusion Criteria

Exclusion criteria were (a) systematic reviews, meta-analyses, letters to the editor, abstracts without a full-text article, and (b) studies with substantial content variations (e.g.*,* influence of UTI treatment on microbiome diversity). (c) All LUTS symptoms due to other comorbidities, such as obesity, renal disorders, diabetes, and bowel disorders, were also excluded, as well as (d) articles not written in English, Dutch, French, or Spanish.

### 2.3. Study Selection and Screening

A search of PubMed, Embase, and CINAHL databases was conducted for the literature published with no publication year restrictions applied. All papers in English, Dutch, French, or Spanish were considered eligible.

Article selection involved evaluating titles and abstracts, with subsequent retrieval and assessment of full-text articles based on pre-established inclusion and exclusion criteria following the PICOS-model (Patient, Intervention, Comparison, Outcome, Study type) [19]. Search strings in chosen databases are shown in Table 1.

Two blinded reviewers (M.V. and L.V.) independently screened, extracted, and reviewed the titles, abstracts, and full texts, using both software’s Rayyan and Silvi*^®^* (Silvi.AI). Discrepancies about article selection from the two authors were resolved by a third reviewer (G.B.).

### 2.4. Data Extraction

For each included study, two authors independently extracted the following data: first author’s last name, publication year, study methodology, method of microbiome analysis, sex distribution, type of microbiome samples analyzed, total number of patients, LUTS of UTI included, number of patients per pathology, mean age of patients, predominant bacteria phylum, class, order, family, genus, and species per group were included, alpha diversity and beta-diversities. When alpha diversity was not reported in full text, key statistical values were systematically extracted from boxplot figures using WebPlotDigitizer, a validated tool designed for accurately converting graphical representations into numerical data [20]. Subsequently, the corresponding standardized mean differences (SMDs) were calculated.

### 2.5. Risk of Bias Assessment

Two authors (M.V. and L.V.) made an independent analysis of the risk of bias using the QUADOMICS checklist, an adaptation of Quality Assessment of Diagnostic Accuracy Studies (QUADAS) for evaluating the diagnostic accuracy of omics-based research [21]. In the case of any difference in scoring the risk, a new evaluation was done by a third author (G.B.). After discussion between the three authors, the consensus was reached that over 50% of the articles met the predefined quality criteria. The QUADOMICS checklist applied in this review can be found in Table A2.

## 3. Results

### 3.1. Study Characteristics and Patient Group Distribution

**Figure 1:**
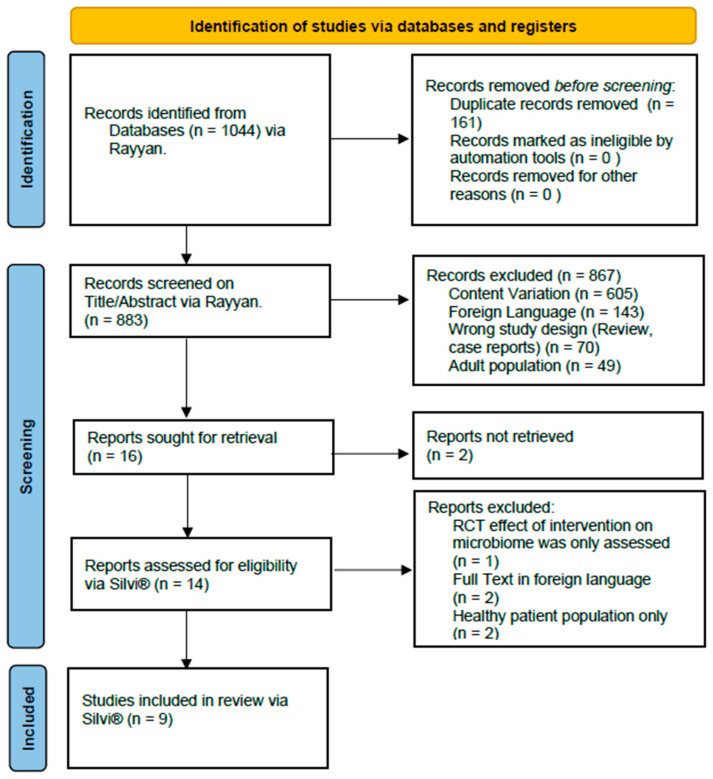
The PRISMA plot of study selection according to the 2020 PRISMA checklist [17].

The PRISMA flowchart is presented in Figure 1: a total of nine studies were included in this systematic review, with publication years spanning from 2018 to 2024. Analysis of the QUADOMCS checklist can be found in Table A3.

Almost all included articles (seven out of nine studies) had a prospective study design, and all studies employed 16S ribosomal RNA sequencing for microbiome analysis, with a combined total of 619 patients. All articles compared microbiome results between cases across various clinical conditions and healthy controls. These clinical conditions included urinary tract infections (UTI), voiding dysfunction (VD), vesicoureteral reflux (VUR), and bladder-bowel dysfunction (BBD). Sample types analyzed included stool in four studies and urine in five studies.

Out of the total 619 patients, 274 patients (44.3%) were part of the pathology groups, while 345 patients (55.7%) were controls. The largest study included 151 patients, while the smallest had 33 participants. The mean patient age varied significantly across studies, ranging from 5 months to 15 years. Both male (38.1%) and female (61.9%) patients were represented.

These study characteristics and patient group distributions are visible in Table 2.

### 3.2. Predominant Bacteria by Sample Type

Predominant bacteria were reported regarding relative abundance between groups in every included article. Both stool and urine samples are separated. Microbiome results are visible in Table 3.

#### 3.2.1. Stool Samples

A total of four studies analyzed stool samples [22,26,28,30]. The predominant bacteria identified from stool samples are summarized below, following clinical conditions:

##### Urinary Tract Infection (UTI)

In patients with UTIs, Actinobacteriota was a predominant identified phylum, followed by *Bacteriodetes* and *Proteobacteria*, with Gram-positive and Gram-negative UTIs having *Enterococcus faecalis* and *Klebsiella pneumoniae*, *Escherichia coli* as predominant species, respectively. Controls typically exhibited a higher prevalence of *Firmicutes* but identically presented *Bacteroidetes*, with genera such as *Bacteroides* and *Veillonella* and species *Bacteroides fragilis* [22,28,30].

##### Voiding Dysfunction (VD)

Specific bacteria identified in stool samples from VD patients included *Fusobacterium nucleatum*, *Clostridium difficile*, and *Bacteroides clarus*, though none had a significant correlation with clinical voiding dysfunction symptom score (VDSS). In controls, *Roseburia intestinalis* was commonly observed [26].

#### 3.2.2. Urine Samples

A total of five studies analyzed urine samples, collected via sterile transurethral catheterization in four articles and in one article via clean-catch midstream method [23,24,25,27,29]. The predominant bacteria identified from urine samples are summarized below, following clinical conditions:

##### Urinary Tract Infection (UTI)

Among UTI patients, families *Enterobacteriaceae*, *Prevotellaceae*, *Veillonellaceae*, and genera *Klebsiella*, *Peptoniphilus*, and *Finegoldia* were more frequently identified in the catheterized urine samples. Family *Neisseriaceae* and genus *Staphylococcus* were more present in control groups [23,24]. History 3 or more UTIs have also shown a decrease in the abundance of genera *Enterococcus*, *Lawsonella*, and *Corynebacterium* [29].

##### Vesicoureteral Reflux (VUR)

Patients with VUR with and without renal scarring exhibited a predominance of genera *Dorea* and *Escherichia* in catheterized samples, whereas controls displayed more *Prevotella* and *Lactobacillus* [25].

**Table 3:** Predominant Bacteria per article.

Publication Year	First Author	Patient Sex Male: Female (*n*:*n*)	Type of Sample	Total *n*	Groups	*n* per Group	Mean Patient Age	Predominant Bacteria					
								Phylum	Class	Order	Family	Genus	Species
2018	Paalanne [22]	30:76	stool	106	UTI	37	20.3 months	*Bacteroidetes,* *Firmicutes*				*Bacteroides,* *Enterobacter*	*Escherichia coli, Bacteroides fragilis, Bacteroides uniformis*
					Control	69	21.8 months	*Bacteroidetes, * *Firmicutes*			*Peptostreptococcaceae*	*Bacteroides*	*Bacteroides fragilis*
2020	Forster [23]	19:15	urine	34	UTI	11	11 years				*Enterobacteriaceae*	*Klebsiella, Staphylococcus*	
					ASB	19	8.8 years				*Enterobacteriaceae*		
					Control	4	15 years				*Enterobacteriaceae, Neisseriaceae*	*Staphylococcus*	
2020	Kinneman [24]	26:59	urine	85	UTI	9	382 days	*Firmicutes, * *Proteobacteria*	*Clostridia, * *Bacteroidia, * *Gammaproteobacteria, * *Actinobacteria, * *Betaproteobacteria*	*Clostridiales, Bacteroidales, Enterobacteriales, Burkholderiales, Actinomycetales*	*Tissierellaceae, Prevotellaceae, Veillonellaceae, Enterobacteriaceae, Comamonadacea*	*Prevotella, Peptoniphilus, Escherichia, Veillonella, Finegoldia*	
					Control	76							
2021	Vitko [25]	12:37	urine	49	VUR	20	4.8 years					*Dorea, Escherichia*	
						13	3.8 years						
					controls	16	10.2 years					*Prevotella,* *Lactobacillus*	
2022	Akarken [26]	20:29	stool	49	VD	25	8.26 years						*Fusobacterium nucleatum, Clostridium difficile,Bacteriodes clarus*
					Control	24	8.00 years						*Roseburia intestinalis*
2023	Cole [27]	0:33	urine	33	BBD	25	8.0 years					*Porphyromonas, Varibaculum, Ezakiella, Campylobacter, Corynebacterium, Dialister, Streptococcus, Escherichia, Lagierella, Schaalia, Lawsonella, Peptoniphilus, Anaerococcus, Lactobacillus, Fenollaria, Finegoldia*	
					Control	8	6.3 years					*Peptoniphilus, Anaerococcus, Lactobacillus, Fenollaria, Finegoldia*	
2023	Urakami [28]	42:37	Stool	79	UTI	28	5 months	*Actinobacceriota, * *Actinobacteria*	*Bacilli*	*Bifidobacteriales, Enterobacteriales*	*Bifidobacteriaceae, Enterobacteriaceae*	*Escherichia, Shigella*	*Escherichia coli*
					Control	51	5 months	*Bacteroidiota*	*Bacteroidia*	*Negativicutes, Bacteroidales, Veillonellases, Selenomonadales*	*Bacteroidaceae, Veillonellaceae*	*Veilonella, Bacteroides*	
2024	Kelly [29]	Male	urine	33	Healthy	13	40.1 months					*Peptoniphillus, Ezakiella, Sphingomonas, Ralstonia*	
		Female				20						*Prevotella, Anaerococcus, Shaalia*	*Prevotella timonensis, Schaalia turincensis,Anaerococcus lactolyticus*
		13:20		33	0 UTI or Unknown (excluded from analysis)	5							
					History of 1 UTI	10							
					History of 2 UTIs	8							
					History of 3+ UTIs	10		*Proteobacteria * *DECREASED:* *Bacteriodetes*				*DECREASED: Enterococcus*, *Lawsonella*, *Corynebacterium*	
2024	Luyang Hong [30]	74:77	stool	151	Gram-positive UTI	53	29.49 weeks		*Gammaproteobacteria, * *Bacilli*		*Enterococcaceae*		*Enterococcus faecalis*
					Gram-negative UTI				*Gammaproteobacteria, * *Bacilli*		*Enterobacteriaceae*	*Klebsiella, Escherichia*	*Escherichia coli, Klebsiella aerogenes,Klebsiella pneumoniae, Enterobacter cloacae*
					Control	98	30.24 weeks		*Clostridia*				

*n*: number of patients; UTI: Urinary Tract Infection; ASB: Asymptomatic Bacteriuria; VUR: Vesicoureteral Reflux; VD: Voiding Dysfunction.

##### Bladder-Bowel Dysfunction (BBD)

Urine samples from BBD patients via the clean-catch method exhibited diverse genera, including *Porphyromonas*, *Varibaculum*, *Ezakiella*, *Campylobacter*, *Corynebacterium*, *Dialister*, *Streptococcus*, *Escherichia*, *Lagierella*, *Schaalia*, and *Lawsonella*. In controls, overlapping genera, such as *Peptoniphilus*, *Anaerococcus*, *Lactobacillus*, *Fenollaria,* and *Finegoldia* were identified [27].

### 3.3. Microbiome Diversity by Sample Type

#### 3.3.1. Stool Samples

##### Alpha Diversity

In stool samples, alpha-diversity indices varied significantly between UTI and control groups. Urakami et al. reported a lower Shannon–Waver diversity index and Chao1 indices in UTI patients compared to controls with calculated standardized mean differences (SMDs) indicating moderate to large effect size differences [28]. Paalanne et al., on the other hand, reported similar indices for alpha diversity in both groups, with calculated SMDs being close to zero [22]. Luyang Hong et al. did not report exact alpha diversity indices, but reported Shannon’s index in the Gram-positive UTI group to be lower than the healthy control group [30].

These results are visible in Table 4.

##### Beta Diversity

Only one article analyzing stool samples reported on beta diversity indices, stating that UTI and control groups formed separate clusters, reflecting significant compositional differences [28].

#### 3.3.2. Urine Samples

##### Alpha Diversity

In catheterized urine samples, decreased alpha diversity in UTI patients (reported with Chao1, Shannon–Waver, or Inverse Simpson Indices) was consistent in multiple articles compared with healthy controls [23,24]. Forster et al. report a significantly lower microbial diversity in UTI patients compared to healthy controls, with large effect sizes (SMD = 1.11–1.54) [23]. Substantial reduction in Shannon entropy (SMD = 3.33) reported by Kinneman et al. in UTI patients compared to non-UTI individuals confirms this major shift in microbial community structure [24]. Similarly, in recurrent UTI patients, a progressive decline in alpha diversity (reported with Chao1 index, Shannon–Waver, and Inverse Simpson indices) was identified, with effect sizes ranging from moderate to large (SMD = 0.58–1.35) [29].

Patients with BBD also exhibited reduced microbial diversity compared to asymptomatic controls (SMD = −0.71), suggesting a potential link between dysbiosis and bladder dysfunction [27]. These urine samples were collected via the clean catch method after professional instruction and assistance in urogenital cleansing.

These results are visible in Table 4.

##### Beta Diversity

Beta diversity analyses (reported with Bray-Curtis and Adonis indices) of catheterized urine samples showed that UTI patients clustered separately from those without UTI [24]. No differences have been reported in BBD patients [27].

## 4. Discussion

This systematic review is the first to evaluate gut and urinary microbiome alterations in pediatric LUTS and UTIs. Findings indicate lower urinary microbiome diversity in UTI patients with transient microbial disruptions. While gut dysbiosis may influence UTI risk, evidence for microbiome alterations in BBD remains inconclusive.

### 4.1. Urinary Tract Infections (UTIs)

Urine microbiome diversity in urine samples was notably lower in UTI patients compared to healthy controls, a finding consistently reported across multiple studies. Reduced alpha diversity, particularly in individuals with recurrent UTIs, suggests that repeated infections and antibiotic exposure may contribute to dysbiosis [29]. Future research should focus on whether identifying shifts in urinary microbiome diversity prior to UTI onset could aid in predicting high-risk individuals, potentially leading to targeted preventative interventions [24]. Moreover, studying the urinary microbiome in isolation does not account for host-microbiome interactions, which may better indicate UTI susceptibility [31].

A promising avenue for UTI prevention involves probiotic-based interventions. For example, probiotic Gram-negative bacteria such as *Escherichia coli* Nissle 1917 have demonstrated antagonistic effects against pathogenic E. coli strains and Pseudomonas aeruginosa infections in animal models [32]. Such approaches could serve as alternatives to traditional antibiotic treatments, reducing the risk of dysbiosis and antimicrobial resistance.

### 4.2. Prior Antibiotic Exposure

Multiple microbiome studies suggest that antibiotic use can significantly impact microbial diversity, though the extent varies based on timing and study parameters. Kinneman et al. found that recent antibiotic use (1–14 days prior to sampling) led to a substantial reduction in species richness (with calculated SMD 0.65, Table 4), while diversity appeared to recover with time, showing minimal differences after 29–60 days (SMD 0.16, Table 4) and near-complete recovery by 61–90 days (SMD 0.04, Table 4) [24]. Dethlefsen et al. also demonstrated that antibiotic treatment led to rapid decreases in taxonomic richness and diversity of the gut microbiome, with only partial recovery weeks after treatment cessation [33]. While focusing on gut microbiota, their temporal analysis provides important parallels for understanding antibiotic effects on other microbial communities.

In contrast, Reasoner et al. reported only a small effect of prior antibiotic use on alpha diversity, with slight increases in Chao1 and Shannon—Wiener indices among antibiotic-exposed individuals (calculated SMDs −0.24 and −0.30, respectively) [9]. Mulder et al. (2019) specifically examined the urinary microbiome following antibiotic exposure, finding significant reductions in Lactobacillus species that persisted for up to 3 months in some patients, potentially explaining the increased susceptibility to UTIs following antibiotic therapy [34]. Additionally, Price et al. observed that women with recurrent UTIs showed lower urinary microbiome diversity even between active infections, suggesting that repeated antibiotic courses may have cumulative effects on microbial communities that extend beyond the immediate treatment period [15].

These findings suggest that while short-term antibiotic use may significantly disrupt microbial diversity, recovery occurs over time. The overall impact on the urinary microbiome may vary depending on the type and frequency of prior antibiotic use, but this is highly dependent on the measurement methods of the microbiome and sampling techniques.

### 4.3. Bladder-Bowel-Dysfunction (BBD)

Although the literature has shown that adult urinary microbiome differs in patients with and without urge urinary incontinence (UUI), no evidence has been found to confirm the hypothesized clinically relevant alterations in the pediatric urobiome associated with BBD [13,35].

### 4.4. Sex-Based Differences in the Urinary Microbiome

The urinary microbiome exhibited notable differences between males and females, particularly around puberty. Storm et al. reported that post-pubertal female urine samples are predominantly enriched with Lactobacillus and Bifidobacterium compared to a different microbial composition in pre-pubertal female samples, with Veillonella, Prevotella, Dialister, Haemophilus, and Schaalia being more abundant. This microbiome shift during puberty is most likely due to hormonal influences during this transition time [35,36]. In contrast, the male urinary microbiome differed less by age, with the only distinguishing detection of Streptococcus oralis in prepubertal males [35]. Interestingly, microbial profiles in prepubertal children resembled those found in adult females, suggesting that the female urobiome establishes a stable composition at puberty and persists into adulthood. Male urobiomes appear to be stable in different age groups [35]. Fredsgaard et al. analyzed the urobiome of asymptomatic children, finding that girls exhibited significantly higher microbial richness and diversity than boys [37]. However, since the study relied on voided samples, the results may reflect urogenital rather than bladder microbiota.

Anatomical differences may also play a role in urobiome diversity. The shorter female urethra may allow for earlier microbial colonization, whereas the longer male urethra may slow the rate of microbial diversification [29]. Contrary to the previous hypotheses, male and female urinary microbiomes differ even before the onset of puberty, with common taxa such as Peptoniphilus and Anaerococcus being highly abundant in both sexes. Kassiri et al. studied the urobiome in healthy prepubertal males with and without prior antibiotic treatment, showing no significant differences in diversity of the microbiome [38]. However, they report greater dissimilarity between the bacterial compositions (PcoA measures) in urine samples of both groups. These findings underscore the need for further research into the developmental, hormonal, and external factors that influence urobiome composition.

### 4.5. The Role of the Gut Microbiome

Emerging evidence links gut dysbiosis to the risk of UTI, with early microbiota changes potentially affecting immune and nervous system development [8]. Urakami et al. propose that interventions aimed at correcting abnormal gut microbiota composition, such as probiotics, prebiotics, and synbiotics, may help mitigate the risk of UTIs in infants [28,39]. Furthermore, longitudinal analysis of faecal calprotectin levels has revealed a decrease preceding UTI onset, suggesting a possible link between gut immunity and UTI susceptibility [30]. Future research should explore how gut microbiota modulation could serve as a preventive strategy for UTIs.

A negative correlation was observed between VDSS and both general bacterial load and Fusobacterium nucleatum counts [26]. Due to the two-way communication between the intestine-brain axis, a potential dysbiosis affects both sides [40,41]. A reduction in the general bacterial load in the patient group with VD could negatively affect autonomic nervous system (ANS) maturation or the coordination between the central nervous system (CNS) and the lower urinary tract.

### 4.6. Limitations

Microbial sequencing methods targeting the V4-V5 region revealed distinct compositions between stool and urine samples. However, the reliability of differential abundance testing methods for low-biomass samples such as urine remains a significant limitation. Current methodologies may be inadequate for distinguishing differentially abundant sequencing features, as highlighted by Reasoner et al. [9]. Furthermore, the absence of several taxonomic families in 16S rRNA sequencing results underscores the methodological limitations of DNA extraction and sequencing approaches, emphasizing the need for complementary techniques to improve urobiome characterization. Heterogeneity in research methodology, including patient age and reporting alpha diversity via different indices in this review, limits the generalizability of these results. Nevertheless, as the literature on the urinary and faecal microbiome linked to LUTS and UTIs in children remains scarce, the articles included in this review remain relevant to the topic. The method of urine sample collection also influences microbiome analysis results, as the urethral passage of urine includes potential added microbiota that are not abundantly present in the bladder. A critical interpretation of these results remains mandatory.

## 5. Conclusions

This systematic review highlights distinct alterations in the urinary and gut microbiomes of pediatric patients with LUTS and UTIs, indicating a lower urinary microbial diversity in UTI patients and potential microbial disruptions linked to recurrent infections and antibiotic exposure. Findings reveal sex-specific differences in the urinary microbiome, with female microbiota composition evolving significantly after puberty. This emphasizes the importance of considering developmental, anatomical, and antimicrobial alterations when investigating the pediatric urinary microbiome. Future research should aim to clarify the functional implications of these microbial shifts, explore their potential as predictive biomarkers, and evaluate microbiome-targeted interventions for the prevention and management of pediatric LUTS and UTIs.

## Figures and Tables

**Table 1:** Search strings in chosen databases.

Database	Search String(s)
PubMed	(“child”[MeSH Terms] OR “pediatrics”[MeSH Terms] OR “Infant, Newborn”[MeSH Terms] OR child*[Title/abstract] OR schoolchild*[Title/abstract] OR infan*[Title/abstract] OR adolescen*[Title/abstract] OR pediatri*[Title/abstract] OR paediatr*[Title/abstract] OR neonat*[Title/abstract] OR boy[Title/abstract] OR boys[Title/abstract] OR boyhood[Title/abstract] OR girl[Title/abstract] OR girls[Title/abstract] OR girlhood[Title/abstract] OR youth[Title/abstract] OR youths[Title/abstract] OR baby[Title/abstract] OR babies[Title/abstract] OR toddler*[Title/abstract] OR teen[Title/abstract] OR teens[Title/abstract] OR teenager*[Title/abstract] OR newborn*[Title/abstract] OR postneonat*[Title/abstract] OR postnat*[Title/abstract] OR perinat*[Title/abstract] OR puberty[Title/abstract] OR preschool*[Title/abstract] OR suckling*[Title/abstract] OR picu[Title/abstract] OR nicu[Title/abstract]) AND (“Urine/microbiology”[Mesh Terms] OR “microbiota”[MeSH Terms] OR “gastrointestinal microbiome”[MeSH Terms] OR “urine microbiome”[Title/abstract] OR (“gastrointestinal”[Title/abstract] AND “microbiome”[Title/abstract]) OR “gastrointestinal microbiome”[Title/abstract] OR (“gut”[Title/abstract] AND “microbiome”[Title/abstract]) OR “gut microbiome”[Title/abstract] OR “microbiota”[Title/abstract] OR “urinary microbiota”[Title/abstract] OR “gut microbiota”[Title/abstract] OR “urine microbiome”[Title/abstract] OR “urine microbiota”[Title/abstract]) AND (“Lower Urinary Tract Symptoms”[MeSH Terms] OR “Nocturia”[MeSH Terms] OR “urinary bladder diseases”[MeSH Terms] OR “Urinary Incontinence”[MeSH Terms] OR “Nocturnal Enuresis”[MeSH Terms] OR “urinary tract infections”[MeSH Terms] OR “pyelonephritis”[MeSH Terms] OR “cystitis”[MeSH Terms] OR “Lower Urinary Tract Symptoms”[Title/abstract] OR “luts”[Title/abstract] OR “Nocturia”[Title/abstract] OR “OAB”[Title/abstract] OR “overactive bladder”[Title/abstract] OR “bed wetting”[Title/abstract] OR “urological symptoms”[Title/abstract] OR “urinary disorders”[Title/abstract] OR “Urinary urge incontinence”[Title/abstract] OR “lower urinary tract dysfunction”[Title/abstract] OR “lower urinary tract problems”[Title/abstract] OR “urinary urgency”[Title/abstract] OR “urinary frequency”[Title/abstract] OR “voiding dysfunction”[Title/abstract] OR (“urinary”[Title/abstract] AND “tract”[Title/abstract] AND “infections”[Title/abstract]) OR “urinary tract infections”[Title/abstract] OR “urinary tract infection”[Title/abstract] OR “UTI”[Title/abstract] OR “UTIs”[Title/abstract] OR “UTIs”[Title/abstract] OR “bacteriuria”[Title/abstract] OR “pyelonephritis”[Title/abstract] OR “cystitis”[Title/abstract] OR “pyuria”[Title/abstract])
Embase	(‘child’/mj OR ‘pediatrics’/mj OR ‘newborn’/mj OR ‘child*’:ti,ab,kw OR ‘schoolchild*’:ti,ab,kw OR ‘infan*’:ti,ab,kw OR ‘adolescen*’:ti,ab,kw OR ‘pediatri*’:ti,ab,kw OR ‘paediatr*’:ti,ab,kw OR ‘neonat*’:ti,ab,kw OR ‘boy’:ti,ab,kw OR ‘boys’:ti,ab,kw OR ‘boyhood’:ti,ab,kw OR ‘girl’:ti,ab,kw OR ‘girls’:ti,ab,kw OR ‘girlhood’:ti,ab,kw OR ‘youth’:ti,ab,kw OR ‘youths’:ti,ab,kw OR ‘baby’:ti,ab,kw OR ‘babies’:ti,ab,kw OR ‘toddler*’:ti,ab,kw OR ‘teen’:ti,ab,kw OR ‘teens’:ti,ab,kw OR ‘teenager*’:ti,ab,kw OR ‘newborn*’:ti,ab,kw OR ‘postneonat*’:ti,ab,kw OR ‘postnat*’:ti,ab,kw OR ‘perinat*’:ti,ab,kw OR ‘puberty’:ti,ab,kw OR ‘preschool*’:ti,ab,kw OR ‘suckling*’:ti,ab,kw OR ‘picu’:ti,ab,kw OR ‘nicu’:ti,ab,kw) AND (‘urine’/mj AND ‘microbiology’/de OR ‘microflora’/mj OR ‘intestine flora’/mj OR (‘gastrointestinal’:ti,ab,kw AND ‘microbiome’:ti,ab,kw) OR ‘gastrointestinal microbiome’:ti,ab,kw OR (‘gut’:ti,ab,kw AND ‘microbiome’:ti,ab,kw) OR ‘gut microbiome’:ti,ab,kw OR ‘microbiota’:ti,ab,kw OR ‘urinary microbiota’:ti,ab,kw OR ‘gut microbiota’:ti,ab,kw OR ‘urine microbiome’:ti,ab,kw OR ‘urine microbiota’:ti,ab,kw) AND (‘lower urinary tract symptom’/mj OR ‘nocturia’/mj OR ‘bladder disease’/mj OR ‘urine incontinence’/mj OR ‘nocturnal enuresis’/mj OR ‘urinary tract infection’/mj OR ‘pyelonephritis’/mj OR ‘cystitis’/mj OR ‘lower urinary tract symptoms’:ti,ab,kw OR ‘luts’:ti,ab,kw OR ‘nocturia’:ti,ab,kw OR ‘oab’:ti,ab,kw OR ‘overactive bladder’:ti,ab,kw OR ‘bed wetting’:ti,ab,kw OR ‘urological symptoms’:ti,ab,kw OR ‘urinary disorders’:ti,ab,kw OR ‘urinary urge incontinence’:ti,ab,kw OR ‘lower urinary tract dysfunction’:ti,ab,kw OR ‘lower urinary tract problems’:ti,ab,kw OR ‘urinary urgency’:ti,ab,kw OR ‘urinary frequency’:ti,ab,kw OR ‘voiding dysfunction’:ti,ab,kw OR (‘urinary’:ti,ab,kw AND ‘tract’:ti,ab,kw AND ‘infections’:ti,ab,kw) OR ‘urinary tract infections’:ti,ab,kw OR ‘urinary tract infection’:ti,ab,kw OR ‘uti’:ti,ab,kw OR ‘utis’:ti,ab,kw OR ‘uti‘s’:ti,ab,kw OR ‘bacteriuria’:ti,ab,kw OR ‘pyelonephritis’:ti,ab,kw OR ‘cystitis’:ti,ab,kw OR ‘pyuria’:ti,ab,kw)
CINAHL/Ebsco HOST	(((MH “Child”) OR (MH “Pediatrics”) OR (MH “Infant, Newborn”) OR (child*) OR (schoolchild*) OR (infan*) OR (adolescen*) OR (pediatri*) OR (paediatr*) OR (neonat*) OR (boy) OR (boys) OR (boyhood) OR (girl) OR (girls) OR (girlhood) OR (youth) OR (youths) OR (baby) OR (babies) OR (toddler*) OR (teen) OR (teens) OR (teenager*) OR (newborn*) OR (postneonat*) OR (postnat*) OR (perinat*) OR (puberty) OR (preschool*) OR (suckling*) OR (picu) OR (nicu))) AND (((MH “Urine/Microbiology”) OR (MH “Microbiota”) OR (MH “Gastrointestinal Microbiome”) OR (urine microbiome) OR (gastrointestinal AND microbiome) OR (gastrointestinal microbiome) OR (gut AND microbiome) OR (gut microbiome) OR (microbiota) OR (urinary microbiota) OR (gut microbiota) OR (urine microbiome) OR (urine microbiota))) AND (((MH “Lower Urinary Tract Symptoms”) OR (MH “Nocturia”) OR (MH “Urinary Bladder Diseases”) OR (MH “Urinary Incontinence”) OR (MH “Nocturnal Enuresis”) OR (MH “Urinary Tract Infections”) OR (MH “Pyelonephritis”) OR (MH “Cystitis”) OR (Lower Urinary Tract Symptoms) OR (LUTS) OR (Nocturia) OR (OAB) OR (overactive bladder) OR (bed wetting) OR (urological symptoms) OR (urinary disorders) OR (Urinary urge incontinence) OR (lower urinary tract dysfunction) OR (lower urinary tract problems) OR (urinary urgency) OR (urinary frequency) OR (voiding dysfunction) OR (urinary AND tract AND infections) OR (urinary tract infections) OR (urinary tract infection) OR (UTI) OR (UTIs) OR (UTIs) OR (bacteriuria) OR (pyelonephritis) OR (cystitis) OR (pyuria)))

**Table 2:** Study characteristics and patient group distribution.

Publication Year	First Author	Study Type	Retrospective vs. Prospective	Method of Microbiome Analysis	Patient Sex Male: Female (*n*:*n*)	Type of Sample	Total *n*	Groups	*n* per Group	Mean Patient Age
2018	Paalanne [22]	case-control	prospective	16S Ribosomal RNA sequencing	30:76	stool	106	UTI	37	20.3 months
								Control	69	21.8 months
2020	Forster [23]	cross-sectional	retrospective	16S Ribosomal RNA sequencing	19:15	urine	34	UTI	11	11 years
								ASB	19	8.8 years
								Control	4	15 years
2020	Kinneman [24]	cross-sectional	prospective	16S Ribosomal RNA sequencing	26:59	urine	85	UTI	9	382 days
								Control	76	
2022	Vitko [25]	case-control	prospective	16S Ribosomal RNA sequencing	12:37	urine	49	VUR without Renal scarring	20	4.8 years
								VUR with Renal scarring	13	3.8 years
								controls	16	10.2 years
2022	Akarken [26]	cross-sectional	retrospective	16S Ribosomal RNA sequencing	20:29	stool	49	Voiding dysfunction	25	8.26 years
								Control	24	8.00 years
2023	Cole [27]	case-control	prospective	16S Ribosomal RNA sequencing	0:33	urine	33	Bladder-Bowel Dysfunction (BBD)	25	8.0 years
								Control	8	6.3 years
2023	Urakami [28]	cross-sectional	prospective	16S Ribosomal RNA sequencing	42:37	Stool	79	UTI	28	5 months
								Control	51	5 months
2024	Kelly [29]	cross-sectional	prospective	16S Ribosomal RNA sequencing	13:20	urine	33	No UTI or Unknown (excluded for analysis)	5	40.1 months
								History of 1 UTI	10	
								History of 2 UTIs	8	
								History of 3+ UTIs	10	
2024	L. Hong [30]	Case-control	prospective	16S Ribosomal RNA sequencing	74:77	stool	151	UTI	53	29.49 weeks
								Control	98	30.24 weeks

**Table 4:** Microbiome alpha diversity per article.

Publication Year	First Author	Patient Sex Male:Female (*n*:*n*)	Type of Sample	Total *n*	Groups	*n* per Group	Mean Patient Age	Alpha Diversity							
								Chao1-Index	SMD	Shannon-Waver	SMD	Inverse Simpson	SMD	Pielou	SMD
2018	Paalanne [22]	30:76	stool	106	UTI	37	20.3 months	1040 (SD 540.5)	−0.02	5.9 (SD 1.61)	−0.13				
					Control	69	21.8 months	1050 (SD 485.0)		6.09 (SD 1.37)					
2020	Forster [23]	19:15	urine	34	UTI	11	11 years	311.38 (SD 140.75)	0.13 ^1^	1.65 (SD 0.44)	−0.23 ^1^				
					ASB	19	8.8 years	156,77 (SD 138.24)	1.54 ^2^	1.34 (SD 1.35)	0.14 ^2^				
					Control	4	15 years	140.34 (SD 100.16)	1.11 ^3^	1.82 (SD 0.98)	0.37 ^3^				
2020	Kinneman [24]	26:59	urine	85	UTI	9	382 days			1.65 (SD 0.44)	3.33				
					Control	76				3.80 (SD 1.58)					
2021	Vitko [25]	12:37	urine	49	VUR	20	4.8 years	Not Reported							
						13	3.8 years								
					controls	16	10.2 years								
2022	Akarken [26]	20:29	stool	49	VD	25	8.26 years	Not Reported							
					Control	24	8.00 years								
2023	Cole [27]	0:33	urine	33	BBD	25	8.0 years	139.03 (SD 81.25)	−0.41	2.51 (SD 1.68)	−0.71				
					Control	8	6.3 years	170.57 (SD 67.70)		3.52 (SD 0.20)					
2023	Urakami [28]	42:37	Stool	79	UTI	28	5 months	42.5 (IQR 33.5–48.5)	1.4	3.0 (IQR 2.7–3.5)	0.77				
					Control	51	5 months	97 (IQR 69.5–132.0)		3.7 (IQR 3.2–4.6)					
2024	Kelly [29]	Male	urine	33	Healthy	13	40.1 months			1.75 (SD 0.94)	0.91	4.30 (SD 2.71)	0.87	0.65 (SD 0.19)	0.57
		Female				20				2.37 (SD 0.43)		7.66 (SD 4.46)		0.73 (SD 0.10)	
		13:20		33	0 UTI or Unknown (excluded for analysis)	5				/		/			
					History of 1 UTI	10				2.58 (SD 0.40)	0.58 ^4^	8.64 (SD 4.34)	0.32 ^4^	0.83 (SD 0.04)	2.38 ^4^
					History of 2 UTIs	8				2.31 (SD 0.55)	0.78 ^5^	7.34 (SD 3.65)	1.14 ^5^	0.70 (SD 0.07)	0.68 ^5^
					History of 3+ UTIs	10				1.62 (SD 1.07)	1.19 ^6^	3.9 (SD 2.43)	1.35 ^6^	0.53 (SD 0.32)	1.29 ^6^
2024	Luyang Hong [30]	74:77	stool	151	Gram-positive UTI	53	29.49 weeks			*Only in the figure*					
					Gram-negative UTI					*Only in the figure*					
					Control	98	30.24 weeks			*Only in the figure*					

SMD: Standardized Mean Difference; SD: Standard Deviation; UTI: Urinary Tract Infection; ASB: Asymptomatic Bacteriuria; VUR: Vesicoureteral Reflux; VD: Voiding Dysfunction; ^1^: SMD between UTI and ASB; ^2^: SMD between control and UTI; ^3^: SMD between control and ASB; ^4^: SMD between 1 UTI and 2 UTIs; ^5^: SMD between 2 UTIs and 3+ UTIs; ^6^: SMD between 1 UTI and 3+ UTIs.

## Data Availability

No new data were created or analyzed in this study. Data sharing is not applicable to this article.

## Abbreviations

The following abbreviations are used in this manuscript:

ANS	Autonomic nervous system
BBD	Bladder-bowel dysfunction
CNS	Central nervous system
LUTS	Lower urinary tract symptoms
NE	Nocturnal enuresis
NGS	Next-generation sequencing
PICOS	Patient, Intervention, Comparison, Outcome, Study type
PRISMA	Preferred Reporting Items for Systematic Reviews and Meta-Analyses
QUADAS	Quality Assessment of Diagnostic Accuracy Studies
QUADOMICS	Adaptation of the QUADAS, studies on the diagnostic accuracy of ‘-omics’-based technologies
rRNA	ribosomal RNA
SMD	Standardized mean difference
UTIs	Urinary tract infections
VD	Voiding dysfunction
VDSS	Voiding dysfunction symptom score
VUR	Vesicoureteral reflux

## Appendix A

**Table A1:** PRISMA 2020 Checklist.

Section and Topic	Item #	Checklist Item	Location Where Item Is Reported
**TITLE**			
Title	1	Identify the report as a systematic review.	p. 1
**ABSTRACT**			
Abstract	2	See the PRISMA 2020 for abstracts checklist.	p. 1
**INTRODUCTION**			
Rationale	3	Describe the rationale for the review in the context of existing knowledge.	p. 1
Objectives	4	Provide an explicit statement of the objective(s) or question(s) the review addresses.	p. 2
**METHODS**			
Eligibility criteria	5	Specify the inclusion and exclusion criteria for the review and how studies were grouped for the syntheses.	pp. 2–3
Information sources	6	Specify all databases, registers, websites, organizations, reference lists, and other sources searched or consulted to identify studies. Specify the date when each source was last searched or consulted.	p. 3
Search strategy	7	Present the full search strategies for all databases, registers, and websites, including any filters and limits used.	pp. 3–4
Selection process	8	Specify the methods used to decide whether a study met the inclusion criteria of the review, including how many reviewers screened each record and each report retrieved, whether they worked independently, and, if applicable, details of automation tools used in the process.	pp. 4–5
Data collection process	9	Specify the methods used to collect data from reports, including how many reviewers collected data from each report, whether they worked independently, any processes for obtaining or confirming data from study investigators, and, if applicable, details of automation tools used in the process.	pp. 4–5
Data items	10a	List and define all outcomes for which data were sought. Specify whether all results that were compatible with each outcome domain in each study were sought (e.g., for all measures, time points, analyses), and if not, the methods used to decide which results to collect.	p. 5
	10b	List and define all other variables for which data were sought (e.g., participant and intervention characteristics, funding sources). Describe any assumptions made about any missing or unclear information.	p. 5
Study risk of bias assessment	11	Specify the methods used to assess risk of bias in the included studies, including details of the tool(s) used, how many reviewers assessed each study, and whether they worked independently, and, if applicable, details of automation tools used in the process.	p. 5
Effect measures	12	Specify for each outcome the effect measure(s) (e.g., risk ratio, mean difference) used in the synthesis or presentation of results.	pp. 2–3 and p. 5
Synthesis methods	13a	Describe the processes used to decide which studies were eligible for each synthesis (e.g., tabulating the study intervention characteristics and comparing against the planned groups for each synthesis (item #5).	pp. 3–5
	13b	Describe any methods required to prepare the data for presentation or synthesis, such as handling of missing summary statistics or data conversions.	N/A
	13c	Describe any methods used to tabulate or visually display the results of individual studies and syntheses.	N/A
	13d	Describe any methods used to synthesize results and provide a rationale for the choice(s). If meta-analysis was performed, describe the model(s), method(s) to identify the presence and extent of statistical heterogeneity, and software package(s) used.	N/A
	13e	Describe any methods used to explore possible causes of heterogeneity among study results (e.g., subgroup analysis, meta-regression).	N/A
	13f	Describe any sensitivity analyses conducted to assess the robustness of the synthesized results.	N/A
Reporting bias assessment	14	Describe any methods used to assess the risk of bias due to missing results in a synthesis (arising from reporting biases).	N/A
Certainty assessment	15	Describe any methods used to assess certainty (or confidence) in the body of evidence for an outcome.	N/A
**RESULTS**			
Study selection	16a	Describe the results of the search and selection process, from the number of records identified in the search to the number of studies included in the review, ideally using a flow diagram.	pp. 5–8
	16b	Cite studies that might appear to meet the inclusion criteria, but which were excluded, and explain why they were excluded.	pp. 5–8
Study characteristics	17	Cite each included study and present its characteristics.	pp. 7–8
Risk of bias in studies	18	Present assessments of the risk of bias for each included study.	Appendix A3
Results of individual studies	19	For all outcomes, present, for each study: (a) summary statistics for each group (where appropriate) and (b) an effect estimate and its precision (e.g., confidence/credible interval), ideally using structured tables or plots.	pp. 5–15
Results of syntheses	20a	For each synthesis, briefly summarize the characteristics and risk of bias among contributing studies.	Appendix A3
	20b	Present the results of all statistical syntheses conducted. If meta-analysis was done, present for each the summary estimate and its precision (e.g., confidence/credible interval) and measures of statistical heterogeneity. If comparing groups, describe the direction of the effect.	N/A
	20c	Present the results of all investigations of possible causes of heterogeneity among study results.	N/A
	20d	Present results of all sensitivity analyses conducted to assess the robustness of the synthesized results.	N/A
Reporting biases	21	Present assessments of risk of bias due to missing results (arising from reporting biases) for each synthesis assessed.	N/A
Certainty of evidence	22	Present assessments of certainty (or confidence) in the body of evidence for each outcome assessed.	N/A
**DISCUSSION**			
Discussion	23a	Provide a general interpretation of the results in the context of other evidence.	pp. 15–17
	23b	Discuss any limitations of the evidence included in the review.	p. 17
	23c	Discuss any limitations of the review processes used.	p. 17
	23d	Discuss implications of the results for practice, policy, and future research.	p. 17
**OTHER INFORMATION**			
Registration and protocol	24a	Provide registration information for the review, including the register name and registration number, or state that the review was not registered.	p. 2
	24b	Indicate where the review protocol can be accessed, or state that a protocol was not prepared.	p. 2
	24c	Describe and explain any amendments to the information provided at registration or in the protocol.	N/A
Support	25	Describe sources of financial or non-financial support for the review, and the role of the funders or sponsors in the review.	p. 18
Competing interests	26	Declare any competing interests of review authors.	p. 18
Availability of data, code and other materials	27	Report which of the following are publicly available and where they can be found: template data collection forms; data extracted from included studies; data used for all analyses; analytic code; any other materials used in the review.	N/A

N/A: Not Applicable.

**Table A2:** Items included in QUADOMICS Checklist.

Items	Possible Answers: Yes/No/Unclear/Not Applicable
1. Were selection criteria clearly described? 2. Was the spectrum of patients representative of patients who will receive the test in practice? 3. Was the type of sample fully described? 4. Were the procedures and timing of biological sample collection with respect to clinical factors described with enough detail? 5. Were handling and pre-analytical procedures reported in sufficient detail and similar for the whole sample? And, if differences in procedures were reported, was their effect on the results assessed? 6. Is the time period between the reference standard and the index test short enough to reasonably guarantee that the target condition did not change between the two tests? 7. Is the reference standard likely to correctly classify the target condition? 8. Did the whole sample or a random selection of the sample receive verification using a reference standard of diagnosis? 9. Did patients receive the same reference standard regardless of the result of the index test? 10. Was the execution of the index test described in sufficient detail to permit replication of the test? 11. Was the execution of the reference standard described in sufficient detail to permit its replication? 12. Were the index test results interpreted without knowledge of the results of the reference standard? 13. Were the reference standard results interpreted without knowledge of the results of the index test? 14. Were the same clinical data available when test results were interpreted as would be available when the test is used in practice? 15. Were uninterpretable/intermediate test results reported? 16. Is it likely that the presence of overfitting was avoided?	

**Table A3:** QUADOMICS Checklist applied to included articles, with individual items scored by every observer, and with total scores.

Article	Observer	QUADOMICS Checklist																QUADOMICS-Score				
		1	2	3	4	5	6	7	8	9	10	11	12	13	14	15	16	1	0	U	N/A	Total
**Paalanne et al. (2018)** [22]	*1*	1	1	1	1	1	1	1	1	1	1	1	U	U	1	0	U	12	1	3	0	12
	*2*	1	1	1	1	1	1	1	1	1	1	1	U	1	1	1	1	15	0	1	0	15
	***Final***	**1**	**1**	**1**	**1**	**1**	**1**	**1**	**1**	**1**	**1**	**1**	**U**	**1**	**1**	**0**	**0**	**13**	**2**	**1**	**0**	**13**
**Forster et al. (2020)** [23]	*1*	1	1	1	1	1	1	1	1	1	1	1	U	U	1	0	U	12	1	3	0	12
	*2*	1	1	1	1	1	1	1	1	1	1	1	U	1	1	1	1	15	0	1	0	15
	***Final***	**1**	**1**	**1**	**1**	**1**	**1**	**1**	**1**	**1**	**1**	**1**	**U**	**1**	**1**	**0**	**0**	**13**	**2**	**1**	**0**	**13**
**Kinneman et al. (2020)** [24]	*1*	1	1	1	1	1	1	1	1	1	1	1	U	U	1	0	U	12	1	3	0	12
	*2*	1	1	1	1	U	1	1	0	1	1	U	1	1	0	U	1	11	2	3	0	11
	***Final***	**1**	**1**	**1**	**1**	**U**	**1**	**1**	**1**	**1**	**1**	**0**	**1**	**1**	**1**	**0**	**0**	**12**	**3**	**1**	**0**	**12**
**Vitko et al. (2021)** [25]	*1*	1	1	1	1	1	1	1	1	1	1	1	U	U	1	0	U	12	1	3	0	12
	*2*	1	1	1	1	1	1	1	1	1	1	1	1	1	1	1	1	16	0	0	0	16
	***Final***	**1**	**1**	**1**	**1**	**1**	**1**	**1**	**1**	**1**	**1**	**1**	**1**	**1**	**1**	**0**	**0**	**14**	**2**	**0**	**0**	**14**
**Akarken et al. (2022)** [26]	*1*	1	1	1	1	1	1	1	1	1	1	1	U	U	1	0	0	12	1	3	0	12
	*2*	1	1	1	1	U	1	1	0	1	1	U	1	1	0	U	1	11	2	3	0	11
	***Final***	**1**	**1**	**1**	**1**	**U**	**1**	**1**	**1**	**1**	**1**	**0**	**U**	**1**	**1**	**1**	**0**	**12**	**2**	**2**	**0**	**12**
**Urakami et al. (2023)** [28]	*1*	1	1	1	1	1	1	1	1	1	1	1	U	U	1	0	1	13	1	2	0	13
	*2*	1	1	1	1	1	1	1	1	1	1	1	U	1	1	1	1	15	0	1	0	15
	***Final***	**1**	**1**	**1**	**1**	**1**	**1**	**1**	**1**	**1**	**1**	**1**	**U**	**U**	**1**	**0**	**1**	**13**	**1**	**2**	**0**	**13**
**Cole et al. (2023)** [27]	*1*	1	U	1	1	1	U	1	1	1	1	1	U	U	1	1	1	12	0	4	0	12
	*2*	1	1	1	1	1	1	1	1	1	1	1	1	1	1	1	1	16	0	0	0	16
	***Final***	**1**	**1**	**1**	**1**	**1**	**1**	**1**	**1**	**1**	**1**	**1**	**1**	**1**	**1**	**1**	**1**	**16**	**0**	**0**	**0**	**16**
**Kelly et al. (2024)** [29]	*1*	1	U	1	1	1	U	U	U	U	1	U	U	U	1	1	1	8	0	8	0	8
	*2*	1	1	1	1	1	1	1	1	1	1	1	1	1	1	1	1	16	0	0	0	16
	***Final***	1	1	1	1	1	1	U	1	U	1	U	1	1	1	1	1	13	0	3	0	**13**
**Luyang Hong et al. (2024)** [30]	*1*	1	1	1	1	1	U	1	1	1	1	1	U	U	1	1	1	13	0	3	0	13
	*2*	1	1	1	1	1	1	1	1	1	1	1	U	1	1	1	1	15	0	1	0	15
	***Final***	**1**	**1**	**1**	**1**	**1**	**1**	**1**	**1**	**1**	**1**	**1**	**U**	**1**	**1**	**1**	**1**	**15**	**0**	**1**	**0**	**15**

Quadomics-Score: ‘1’: Item is described, ‘0’: item is not described, ‘U’: Item is described unclearly, ‘N/A’: Item is not applicable.
